# Immunometabolic regulation during the presence of microorganisms and parasitoids in insects

**DOI:** 10.3389/fimmu.2023.905467

**Published:** 2023-09-25

**Authors:** Shirong Li, Jing Wang, Xing Tian, Shahzad Toufeeq, Wuren Huang

**Affiliations:** ^1^ Key Laboratory of Insect Developmental and Evolutionary Biology, CAS Center for Excellence in Molecular Plant Sciences, Shanghai Institute of Plant Physiology and Ecology, Chinese Academy of Sciences, Shanghai, China; ^2^ College of Life Sciences, Yan’an University, Yan’an, Shaanxi, China; ^3^ College of Life Sciences, Shangrao Normal University, Shangrao, China

**Keywords:** insects, *Drosophila*, immunometabolism, pathogenic, symbiotic bacteria, insulin signaling, IMD/Toll

## Abstract

Multicellular organisms live in environments containing diverse nutrients and a wide variety of microbial communities. On the one hand, the immune response of organisms can protect from the intrusion of exogenous microorganisms. On the other hand, the dynamic coordination of anabolism and catabolism of organisms is a necessary factor for growth and reproduction. Since the production of an immune response is an energy-intensive process, the activation of immune cells is accompanied by metabolic transformations that enable the rapid production of ATP and new biomolecules. In insects, the coordination of immunity and metabolism is the basis for insects to cope with environmental challenges and ensure normal growth, development and reproduction. During the activation of insect immune tissues by pathogenic microorganisms, not only the utilization of organic resources can be enhanced, but also the activated immune cells can usurp the nutrients of non-immune tissues by generating signals. At the same time, insects also have symbiotic bacteria in their body, which can affect insect physiology through immune-metabolic regulation. This paper reviews the research progress of insect immune-metabolism regulation from the perspective of insect tissues, such as fat body, gut and hemocytes. The effects of microorganisms (pathogenic bacteria/non-pathogenic bacteria) and parasitoids on immune-metabolism were elaborated here, which provide guidance to uncover immunometabolism mechanisms in insects and mammals. This work also provides insights to utilize immune-metabolism for the formulation of pest control strategies.

## 1 Introduction

Microbes and animals have a long-term association that is widely distributed in nature, and plays a vital role in animal adaptation and evolution. Immunity and metabolism are two different biological systems that have traditionally been studied independently, however, recent studies discover that these two processes are interlinked in animal physiology, and has opened up an exciting area of research called immunometabolism (1). More than a century ago, Elie Metchnikoff one of the founders of immunology, observed metabolic changes associated with inflammation phenotypes (2). Further studies have revealed that many metabolic diseases including type 2 diabetes and obesity are partly because of chronic inflammation further demonstrating the importance of immune-metabolic interactions in animal physiology (3–5). The occurrence of an immune response is an energy consuming process. When organisms are infected with pathogens, the immune system is activated, which is closely related to the body’s metabolic switch, including the redistribution of energy resources and increase of glycolysis and glucose consumption by the immune system (6, 7).

Immune-metabolic interactions are an evolutionary conserved phenomenon in all multicellular organisms from vertebrates to invertebrates, and studies have found similar immune-metabolic phenotypes. For example, *Drosophila melanogaster* infection with parasitic wasps or bacteria results in delayed development and increased carbohydrate mobilization resulting in hyperglycemia-like phenotype, while the energy requirements of immune cells increase from 10% of total glucose consumption to nearly one-third (8, 9). Furthermore, infection induces activation of immune responses leads to a decrease in the overall metabolic rate (10–13), and slow host development and loss of energy reserves during chronic infection (9, 14). Despite the important impact of immune-metabolic interactions on the physiology of the organism, there is currently a large gap in understanding of immune-metabolic interactions. Here, we focus on the metabolic regulations during immune responses in insects, from *D. melanogaster* a genetically tractable insect model to other important agricultural insects. In view of bacteria and parasitic wasps which are two major groups interacting with insects, this paper reviews the research progress of immunometabolic regulation in their interactions with insects. In addition, some classical studies on the effects of parasites on immunometabolism of insects are also included. 

## 2 Metabolic regulation

**Figure 1:**
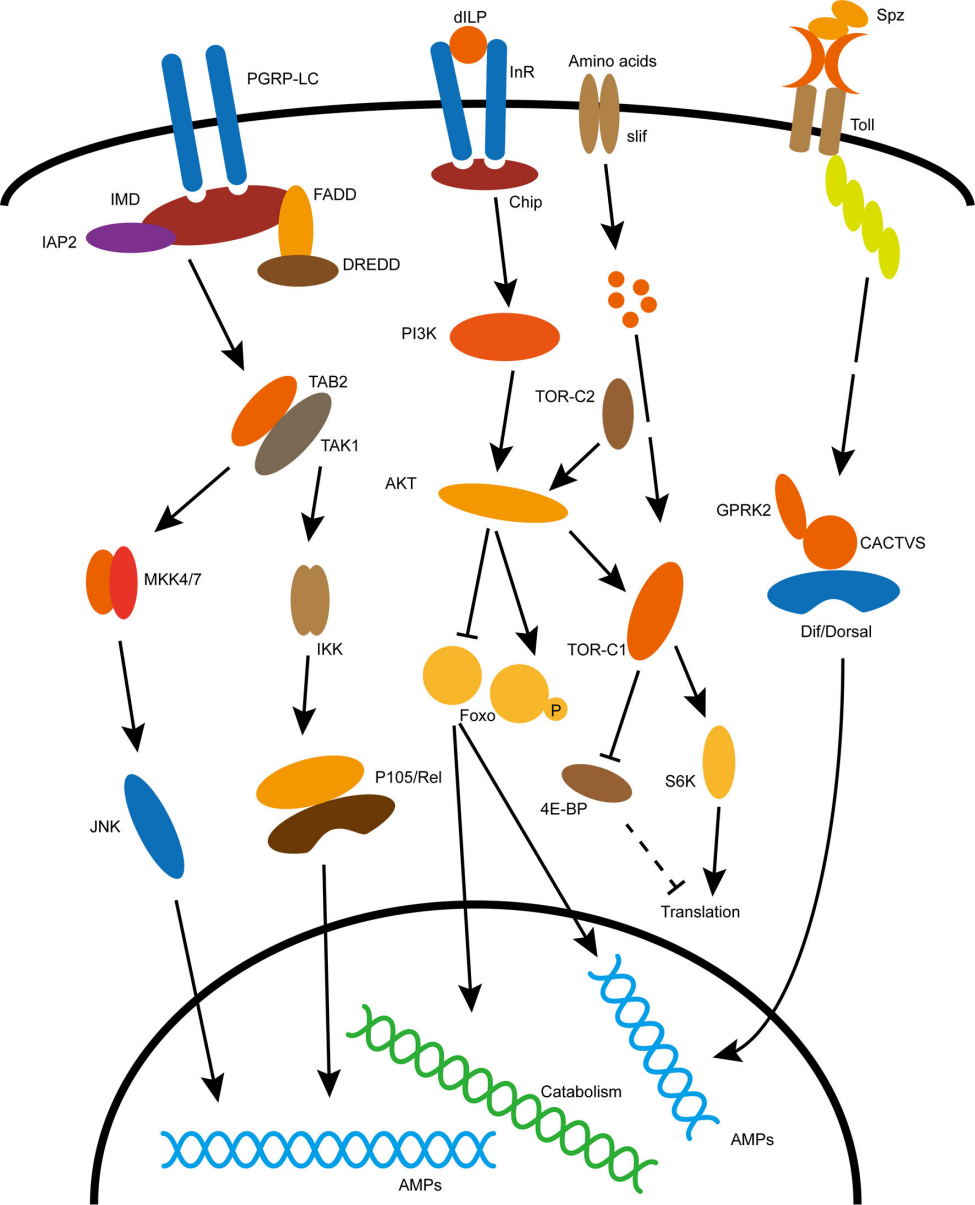
Classical immune and metabolic signaling pathways in insects. Specific explanation is described in the text. The acronyms in the picture are explained below: dILP, Drosophila insulin like peptide; InR, insulin receptor; Chip, insulin receptor substrate; PI3K, phosphoinositide 3-kinase; AKT, Akt kinase; TOR, target of rapamycin; FoxO, forkhead box O transcription factors; Slif, Amino acid transporter; S6K, phosphorylates S6 Kinase; 4E-BP,eIF4E-binding protein; Spz, Spätzle, ligand for Toll pathway activation; Toll, recepter of Toll pathway; Dif/Dorsal, transcription factor of Toll pathway; PGRP-LC, recepter of IMD pathway; IMD, canonic component of the immune deficiency pathway; Rel, transcription factor of IMD pathway; JNK, c-Jun N-terminal kinase cascade.

Organisms adapt to changes in external and internal environments through various metabolic regulation. Insulin/IGF signaling (IIS) is a core pathway regulating the balance between anabolic and catabolic processes in the body (15). IIS activity is systematically regulated by insulin-like peptides (ILPs). *D. melanogaster* contain eight ILPs (DILP1-8), however, there are only two known receptors, dInR (Drosophila insulin receptor) and Lgr3 (leucine-rich G-protein-coupled receptor) for them (16–20). Activited dInR promotes a conserved intracellular signaling cascade transmitted through Chico, a homologue of mammalian insulin receptor substrates (IRS1-4) (21). Together with phosphatidylinositol-3-kinase (PI3K) and protein kinase B (PKB or AKT), IIS affects gene expression through AKT-mediated phosphorylation of FoxO, and subsequent cytoplasmic retention of FoxO, leading to downregulation of its target genes (22–24) (**Figure 1**). Without immune stimulation, insulin signaling cascade regulates energy and nutrient use for storage, growth and other non-immune processes. The inhibition of IIS has been established as an evolutionary conserved mechanism that promotes diapause in invertebrates and prolong lifespan in both invertebrates and vertebrates in response to environmental challenges (25, 26). The mutation of InR substrate Chico flies with suppressed insulin activity, not only extend longevity when live in normal condition but increase pathogen resistance when infected by Gram-negative bacteria *Pseudomonas aeruginosa* and gram-positive bacteria *Enterococcus faecalis (* 27). However, when infected with the insect-pathogenic *Photorhabdus luminescens* and non-pathogenic *Escherichia coli*, there was no apparent difference in survival between Chico mutant flies and controls, furthermore, after infection Chico mutant flies had lower bacterial loads at most time points (28). In addition, transcriptional sequencing analysis of the insulin-resistant fat body of *D. melanogaster* also revealed a correlation between IIS and immune response (29).

## 3 Insect innate immunity

Insects occupy a wide range of ecological niches where they inevitably face frequent contact with microorganisms in the surrounding environment, a small number of which can cause diseases. Although, insects lack an adaptive immune system similar to that of vertebrates, they have a variety of ways to fight off infection by pathogens. The first line of defense in insects depends on behavioral adaptations and body barriers; Behavioral adaptations include insect hygienic behaviors, reduced social contacts, and selective foraging; Body barriers include exoskeleton, trachea and intestinal epithelium and intestinal peritrophic matrix (30–32). The innate immune system is able to distinguish between self and non-self. Insects are able to detect different types of microorganisms by recognizing microbe-associated molecular patterns (MAMPs) through pattern recognition receptors (PRRs) (33). Insect innate immune system contain two different types of immune responses, mainly humoral responses including secretion of antimicrobial effectors such as antimicrobial peptides (AMPs) and lysozyme, and cellular responses such as phagocytosis, encapsulation and nodulation (33). Insect cellular response mainly mediated by hemocytes which contains different types of cells responsible for distinct functions. The *D. melanogaster* larvae contains three major hemocyte cells including plasmatocytes, crystal cells, and lamellocytes that participate in phagocytosis, PO cascade, and encapsulation, respectively (33). While the humoral immunity is mainly regulated by Toll and immunodeficiency (IMD) pathways (33–35). The *D. melanogaster* Toll and IMD pathways share significant similarities with the mammalian TNF-α pathway and the Toll-like receptor pathway (TLR), respectively (36–38). The IMD pathway activates in immune tissues throughout the body, and recognizes diaminopimelic acid (DAP) type peptidoglycans present on cell walls of most Gram-negative bacteria and some Gram-positive bacteria (39). In contrast, the Toll pathway activates primarily in the fat body or hemolymph or immune cells, and respond to MAMPs from Gram-positive and fungi containing lysine-type peptidoglycans and *β*-glucans, respectively (40). In response to pathogen infection, the Toll and IMD signaling pathways of *D. melanogaster* activate the p65-like transcription factor Dif and p105-like transcription factor Relish, respectively; The transcription factor then enters the nucleus and induces the expression of antimicrobial peptide (AMP) by binding to the κB binding site in the AMP gene promoter region (33, 36, 41) (**Figure 1**).

During non-infectious stage, the immune system remains inactivate, consuming the minimum energy for basic functioning (42). These immune responses during pathogenic infection require timely and precise energy allocation in appropriate cells or tissues for effective self-protection and elimination of pathogens (1). Activation of immune system consumes substantial amount of energy and therefore competes for energy resources needed by other biological processes in the body and this energy competition is well documented in eco-physiological studies of insects (43). Present studies on immunometabolism of insects are mainly in the gut, fat body and hemolymph. This is elaborated below.

## 4 Gut

The gut is the most important part of the insect digestive system, and the insect gut epithelium is generally considered to be the only way for insects to obtain nutrients (44, 45). On the one hand, gut coordinates defenses against microbial penetration of the intestinal epithelium, on the other hand, it uses digestive enzymes and transporters to transport nutrients to the internal organs (46). Therefore, the link between metabolism and immunity is especially evident in the gut.

### 4.1 Gut structure and function

In contrast to many multicellular organisms, the intestinal epithelium of insects is simple, consisting of a single cell layer surrounded by a layer of muscle, and in most insects includes only two differentiated cell types: the enterocytes and the endocrine cells (47). The midgut is the main site for food digestion, nutrient absorption, and energy substrate storage (45). The *D. melanogaster* midgut is composed of four different types of cells, including intestinal stem cells (ISCs) and undifferentiated ISC daughter cells referred as enteroblasts (EBs) (48), which can differentiate into intestinal enterocytes (ECs) or enteroendocrine cells (EEs) (49–51). ISCs usually occur singly or in small groups and are often located in the basal lamina (50, 52). Some midgut cells of other insects also include cells with specialized functions for ion transport, such as goblet cells of Lepidoptera and cuprophilic cells of Diptera, which have the function of maintaining highly alkaline and acidic conditions in the intestinal lumen, respectively (53, 54).

In addition, the insect gut has the peritrophic membrane as its physical barrier, establishing a first line of defense against pathogens, and preventing them from contacting intestinal epithelial cells (55). The peritrophic membrane is a rectangular grid-like structure composed of chitin polymers and proteins such as peritrophin (56, 57). The peritrophic membrane acts like a sieve, limiting the passage of not only pathogens but also toxins and food particles (57). In vertebrates, a mucus layer composed of polysaccharides and proteins (mucins) separates the intestinal epithelium from the outside environment, and the mucus layer keeps bacteria confined to the intestinal lumen (58). The peritrophic membrane of insects acts like a vertebrate mucous layer. In *D. melanogaster*, the peritrophic membrane is lined up along the epithelial cells of the midgut, just like a mucous layer (59). Annotated information of more than 30 *D. melanogaster* genomes indicated the presence of genes encoding mucilage layer proteins (60). With the development of mucilage material staining technique (cyclic acid Schiff method), the mucilage layer of *D. melanogaster* was identified (58), but its function remains to be further studied. Transcriptomic data show that genes for both peritrophic membrane metabolism and mucus production are altered in the midgut of Erwinia carotovora subsp. carotovora 15 (Ecc15) infected flies, suggesting that both barriers are remodeled during infection (61).

### 4.2 Gut immunity

Current research on gut immunity mainly focuses on the IMD signaling pathway and the ROS production. The production of reactive oxygen species (ROS) is the first immune response against pathogens in gut, and activated by dioxygenase (DUOX), a member of the nicotinamide adenine dinucleotide phosphate oxidase (NADPH) family (62). Bacterial-derived uracil can induce the formation of calcineurin 99C (Cad99C)-containing endosomes that serve as a phospholipase Cβ (PLCβ)-dependent calcium mobilization platform that is critical for DUOX activation (63). In the midgut, the production of AMPs is regulated by the IMD pathway. The IMD pathway in the gut is activated by two upstream receptors, the cell membrane surface-bound receptor PGRP-LC which acts in the foregut, midgut and hindgut and the intracellular receptor PGRP-LE acts only in the midgut (64, 65). PGRP-LC and PGRP-LE can recognize diaminopimelic acid (DAP) type peptidoglycans present on cell walls of most Gram-negative bacteria and some Gram-positive bacteria (66, 67). When the IMD pathway in the gut is triggered by pathogenic bacteria, intestinal enteroblast ECs release humoral immune factors such as AMPs (47).

### 4.3 Regulation of gut’s immunometabolism caused by pathogenic bacteria

The pathogenic bacteria can affect gut homeostasis by directly disrupting epithelial tissue, or indirectly by altering the structure of gut microbial populations (58, 68). Therefore, maintenance of gut homeostasis requires the synergy of antimicrobial and metabolic responses in order to fight pathogenic infection. Recently, the link between the production of ROS and metabolism have been reported by Lee et al. (69). Their study found that the activation of DUOX is depended on tumor necrosis factor (TRAF3). Further, the activation of TRAF3 can inactivate AKT and activate AMPK, and the inactivation of AKT leads to decrease TOR activity then influence the activity of downstream kinases S6K and ATG1 (69). ROS production can lead to S6K inactivation and ATG1 activation, inactivated S6K resists anabolism, and activated ATG1 promotes catabolism, thereby regulating the transition from lipogenesis to lipolysis in the gut epithelia (69). The maintenance of DUOX activity relies on lipolysis to increase NADPH production, thus, the interaction of lipolytic signaling with host immune signaling ensures an effective antimicrobial response (69). When insect-pathogenic bacteria *Pseudomonas entomophila* infects *D. melanogaster*, the production of bacterial’s toxins and host’s ROS can inhibit TOR pathway and cause complete termination of protein synthesis, thereby preventing tissue repair and antimicrobial peptide production, ultimately accelerating infection to hosts death (70). Wang et al. showed that *Plasmodium falciparum*, the deadliest parasite in humans, could induce activation of P38-MAPK in the midgut of its insects vector *Anopheles stephensi* to weaken the insect’s immune system including inhibition the production of mitochondrial ROS and in turn, enhancing protein synthesis and metabolism (71). Therefore, the triggered p38-MAPK signal in the gut of *A. stephensi* contributes to both the survival of the parasite and avoids the damage of the host by immune overreaction (71).

The two-component system of *Vibrio cholerae* contains carboxylic acid regulator and sensor CrbRS which activates transcription of gene acs1 encoding Acetyl-CoA synthase (ACS-1) to deplete acetate in the gut (72, 73). Oral infection with *Vibrio cholerae* induces intestinal acetate depletion in *D. melanogaster*, systemically inhibits the insulin pathway, promotes fat accumulation in intestinal ECs cells, and accelerates infection-induced host death (73). IMD mutant flies have significantly improved survival after oral infection with *V. cholerae* compared to wild-type, and this phenotype is dependent on the interaction of *V. cholerae’s* type 6 secretion system (T6SS) and the gut commensal bacteria *Acetobacter pasteurianus* to cause immune hyperactivity of wild-type hosts (74). The systemic infection by non-pathogenic bacteria *Escherichia coli*, the extracellular bacterial pathogen *Photorhabdus luminescens*, and the facultative intracellular pathogen *Photorhabdus asymbiotica* cause accumulation of lipid droplets in the midgut of *D. melanogaster* (75). The specific mechanism is that systemic infection leads to mediate the midgut IMD activation through the receptor PGRP-LC, which in turn leads to a decrease in the synthesis of tachykinin TK, which ultimately leads to an increase in midgut fat anabolism; Deliberately reducing TK synthesis in gut can increase the survival time of host under *P. asymbiotica* infection, but make host more sensitive to *P. luminescens* infection (75). In addition, oral infection with *V. cholerae* can also cause lipid metabolism disorders in the host gut and accumulation of triacylglycerols (TAGs) and sterols in intestinal epithelial cells of enterocytes (44).

In *Anopheles gambiae*, phenylalanine hydroxylase (PAH) which is involved in the conversion of phenylalanine to tyrosine, is a key pathway in amino acid metabolism, and it has been found that silencing of PAH destroys phenylalanine metabolism, resulting in a decrease in tyrosine content, leading to a marked impairment of the gut melanin-dependent encapsulation response to the rodent malaria parasite *Plasmodium berghei* (76, 77). These studies indicate that when pathogenic microorganisms invade the gut, the energy demand for immune responses increases to resist microbial invasion by coordinating with the IIS and TOR pathways.

### 4.4 Effects of gut commensal microbiota on host immunity and metabolism

The animal gut plays vital role to interact with the microenvironment. Gut-microbe interactions can have important effects on the host, both through microbial-associated molecular patterns (MAMPs) on microbial surfaces and through the metabolites they produce (78, 79). Although members of the microbiota are often referred to as commensals, symbiosis between the microbiota and its host encompasses various forms of relationship, including mutualistic, parasitic, or commensal (80). The presence of gut commensal microbes is essential for establishment and maintenance of immune system of multicellular organisms (80–82). Moreover, gut microbes also have important effects on the metabolic processes of their host animals, such as directly providing the host with essential nutrients (such as vitamins B and K) or indirectly fermenting indigestible carbohydrates, as well as enhancing the activity of host metabolic pathways (78, 79, 83, 84). The study showed that germ-free mouse epithelial cells expressed fewer metabolic key enzymes than normal mice, and significantly down-regulated nicotinamide adenine dinucleotide (NADH)/NAD^+^ and ATP levels illustrating gut microbes affect epithelial metabolism (85). Several studies using 16S rRNA analysis to determine the common bacterial diversity of *D. melanogaster* showed that both wild and laboratory-raised populations exhibited low bacterial diversity (1-30 species) in the gut (48, 86–88). Furthermore, these bacteria are not essential for host development or survival, as germ-free *D. melanogaster* can be maintained for generations under adequate dietary conditions in the laboratory (89).

The recognition of gut commensal bacterium *Lactobacillus plantarum* (WJL) by the IMD pathway induces intestinal peptidase expression, and elevated levels of intestinal peptidase promote the body to digest proteins, increase the host’s amino acid levels, and promote the TOR pathway in *D. melanogaster* larvae (90, 91). In line with this, transcriptional studies on germ-free, IMD mutants and enteric IMD-constitutively activated *D. melanogaster* confirmed the effects of IMD on intestinal metabolic processes, including the expression of digestive peptidase (92–95). In addition, *L. plantarum* can promote host growth by secreting N-acetyl-glutamine as a byproduct, and hosts in turn improve the environmental adaptability of their symbionts (96, 97). The studies found that the intestinal innate immune NF-kB/Relish transcription factor is a key point for coupling nutrition-immunity-metabolism, Relish regulates the association between diet and host-*Lactobacillus* by restricting 4E-BP/Thor, adjusting the species composition of gut microbes (96–98). *Acetobacter pomorum*, another major gut commensal bacterium of *D. melanogaster*, could promote the growth of larval and adult by increasing the insulin signaling (IIS) pathway (99). Specifically, *A. pomorum* produces acetic acid via alcohol dehydrogenase (PQQ-ADH), a bacterial-derived short-chain fatty acid that affects health and homeostasis in many biological models (100–104). The increase in intestinal acetate levels activate signaling through the host IIS cascade, affecting host growth, energy metabolism, and intestinal stem cell activity (99). Additionally, microbial-derived acetate can activate the IMD pathway of enteroendocrine cells, and the enhancement of IMD signaling can promote the expression of tachykinin (TK) gene to increase the content of TK (105). TK plays an important role in larval growth and development, lipid metabolism and insulin signal transduction (105–107). Sannino et al. showed that the essential vitamin thiamine provided by *A. Pomorum* is essential for the growth and development of *D. melanogaster* (108). Previous work showed that germ-free *D. melanogaster* store more triglyceride than conventionally reared flies and this phenotype can be rescued by recolonization of *Acetobacter fabarum* or *Lactobacillus brevis* (109, 110). The population structure of symbionts in wild *Drosophila* species are further complicated by differences in geographical populations and food sources (88, 111–114). A study showed that *Acetobacter* bacteria isolated from wild *D. melanogaster* can stably colonize in the gut of lab stock and promote larval growth (115). Therefore, the gut commensal microbes, as an additional source of systemic or local immune signals, may be directly involved in regulating the energy homeostasis and growth metabolism of the organism.

Microbe-associated molecular patterns (MAMPs) also exist in commensal microorganisms, and can be recognized by the host immune system (116). The cell walls of both *Acetobacter* and *Lactobacillus*, which dominate the gut flora of *D. melanogaster*, contain DAP-type peptidoglycan, therefore, can be recognized by the gut immune responses (86). Moreover, it was found that the expression AMPs was significantly reduced in germ-free *D. melanogaster* gut, suggesting that commensal microbiota can induce the IMD pathway (68, 86). PGRP-SC2 is a negative regulator of the *D. melanogaster* IMD pathway and is homologous to the vertebrate anti-inflammatory proteins PGLYRP1-4 (117, 118). A study reveal that decreased expression of PGRP-SC2 leads to activation of the IMD transcription factor Relish, resulting in altered commensal microbial community composition and increased division of ISCs (119). These studies demonstrate the importance of IMD pathway activity for the composition of gut commensal microbiota.

Duox is a member of the NADPH oxidase family whose activity can be partially activated in the gut by commensal microorganisms and food-derived yeasts, respectively (62, 120). In the presence of gut commensal microbiota, the activity of p38-MAPKs is down-regulated by MAPK phosphatase 3 (Mkp3), thus attenuating Duox activity and maintaining it at a low-level (121). Interestingly, the intestinal epithelial cells of *Drosophila* contain a second NADPH oxidase, the nitrogen oxidase (Nox), which can be activated by gut commensal bacteria *Lactobacillus* to produce ROS as a local signaling molecule, which had no deleterious effects on both bacteria and intestinal epithelium cells (122).

Although commensal and pathogenic microorganisms involve in activation of similar gut immune mechanisms, the level of immune response activated by the intestinal commensal microbiota and the degree of damage to intestinal epithelial cells were much lower than that of pathogenic microorganisms (61). Gut commensal microbiota can induce innate immune signals in intestinal epithelial cells, while negative feedback mechanisms limit the expression of immune factors such as AMPs and ROS that regulate bacterial composition in the gut lumen (86, 121, 123). Honeybee (*Apis mellifera*) exposure to a certain concentration of neonicotinoid insecticide (nitenpyram) causes imbalance of gut microbiota that leads to the alternation of metabolism and immune-related genes in the gut (124). These findings reveal that the insect gut immune system not only eliminate pathogenic microbes but also maintain commensal microbes to regulate metabolism. Therefore, the gut commensal microbes properly coordinate with the host’s immunity and metabolism establishing a long microbiota-insect evolutionary interaction.

## 5 Cross-talk of humoral immunity with metabolism in fat body

Insect fat body is the major organ that provide energy for metabolism and induce humoral immune responses. On the one hand, it is responsible for storing energy including glycogen, triglycerides etc., on the other hand, it provides energy essential for all life activities including immune responses (33, 125). Under the influence of a pathogenic infection, the fat body initiates humoral immune responses including production of AMPs and other immune molecules, restricting energy flow to other processes including anabolism (126).

The fat body’s function of *D. melanogaster* is analogous to mammalian liver, adipose tissues and immune organs (127, 128), it provides a simple and accessible system for studying molecular integration of immune and metabolic pathways in the presence of microorganisms. In *D. melanogaster*, metabolism and growth is regulated by insulin-like peptides that bind to insulin receptors on the fat body cells, activate AKT and TOR, and promote protein synthesis and storage of sugar as triglycerides and glycogen in the fat body (125). Fat body responds to systemic infection of different pathogens through Toll and IMD pathways, driving expression of AMPs to eliminate pathogens (34, 35). Additionally, Eiger/TNF-α, JNK and JAK-STAT are also important immune signals in the fat body in response to various immune stimuli (129). These immune signaling pathways are activated upon infection and regulate a wide range of immune response as well as affect metabolism at various levels. Therefore, they can be considered as key components of immune-metabolism integration. Keeping in view the significance of fat body in immunometabolism, several key pathways are discussed below.

### 5.1 IMD and Toll

IMD pathway is an important NF-κB signaling pathway in the fat body immune regulation. Over-expression of the IMD transcription factor Relish in the fat body does not affect insulin activity (130). However, over-expression of the active form of IMD protein in the fat body suppresses systemic IIS activity and exhibits many phenotypes similar to insulin loss-of-function (131). MEF2 (Myocyte enhancer factor 2) plays a bidirectional regulatory role in fat body anabolism and catabolism through phosphorylation and dephosphorylation (132). MEF2 mediates a metabolic switch under conditions of Gram-negative bacterial infection, shifting the fat body from anabolism to activation of IMD signaling (131). Under normal condition, phosphorylated MEF2 can activate the expression of genes involved in anabolic processes, however, infection leads to dephosphorylation of MEF2, which reduces anabolic processes, while dephosphorylated MEF2 can activate the expression of AMPs (131).

DiAngelo et al. performed selective activation of the Toll signaling pathway in the fat body using genetics and infection to induce immunity and energy redistribution in *D. melanogaster* (130). The study revealed that activation of Toll signaling in the fat body not only decrease the levels of AKT phosphorylation and attenuated insulin signaling in the fat body, but also suppresses insulin signaling throughout the body resulting in decreased nutrient storage and growth retardation (130). Overexpressing the antimicrobial peptide Drosomycin that responds to the Toll pathway reduced glycogen and triglyceride storage of *D. melanogaster* (133), and this suggests that inducing the expression of AMPs may indeed cause the body’s energy burden. Activation of Toll in the fat body reduces triglyceride storage and has systemic effects on body growth, suggesting that immune activation in the fat body can lead to a systemic metabolic switch by reducing insulin signaling (129, 132). Roth et al. further showed that growth inhibition induced by overexpression of Toll in the fat body could be rescued by the expression of phosphorylated AKT (134). Activation of Toll signaling in the fat body of *D. melanogaster* larvae can cause a shift in lipid metabolism, shifting fatty acids from neutral lipid storage to phospholipid synthesis, a shift that supports immune responses in the short term but increases host mortality in the long term (135). Furthermore, the activation of fat body Toll signaling by genetic or bacterial stimulation results in not only tissue-autonomous reductions in triglyceride storage, but the membrane phospholipid synthesis is induced, as manifested by increased levels of phosphatidylcholine and phosphatidylethanolamine in the fat body, this shift facilitates the synthesis and secretion of AMPs (134). Additionally, Toll activation has also been shown to block S6K-mediated phosphorylation of MEF2, leading to a switch from anabolism to immunity in the fat body (132).

From *Drosophila* to mammals, Hippo signaling pathway plays a vital role in regulating tissue growth, organ size and homeostasis (136–138). In mammals, the Hippo signaling pathway is involved in the regulation of islet cells. Inactivation of Hippo in islet cells can damage islet cells and promote their apoptosis, thereby affecting insulin secretion, causing metabolic disorders and dysfunction, and leading to the occurrence of diabetes (139, 140). Liu et al. demonstrated that the Hippo signaling pathway has an important role in response to Gram-positive bacterial infection in *D. melanogaster* and that Hippo activation in the fat body can promote the expression of AMPs (141). Activation of the hippo-Yorkie signaling pathway through the Toll-Myd88-Pelle cascade can induce the degradation of the subunit Cka of the Hpo-inhibitory complex, and then Warts-mediated Yorkie inactivation increases Dif activity by limiting Cactus levels and enhances AMPs gene expression (141). Aforementioned results manifest a strong relationship between the Toll signaling pathway and the growth metabolism pathway: IIS and hippo pathway in the immune metabolism of the fat body.

### 5.2 JNK

JNK (c-Jun N-terminal kinase) signaling can affect metabolism and immunity in many different ways (43). As a stress and inflammatory signaling pathway, JNK signaling can systemically antagonize IIS by activating FoxO to down-regulate the expression of Dilp2 in IPCs (insulin-producing cells), promoting stress tolerance and prolonging lifespan (71). Moderate JNK signaling activity facilitates the management of energy resources under stressful conditions, but excessive JNK activity in vertebrate adipose tissue has been found to cause type II diabetes (142–144). Therefore, tight regulation of the interaction between the stress signal JNK and the insulin signal (IIS) is necessary for the body to ensure its own homeostasis to adapt environmental challenges. Activation of JNK signaling in the fat body can compensate for the cytoplasmic retention of FoxO caused by IIS overexpression, allowing FoxO to enter the nucleus to regulate target gene expression and antagonize IIS-mediated cell overgrowth (145). *Drosophila* NLaz is a secreted protein homologous to vertebrate apolipoprotein D (ApoD) and retinol binding protein 4 (RBP4) (146). Oxidative stress or activation of JNK can induce the transcription of NLaz gene. NLaz mediates the antagonism between JNK and Insulin signaling to negatively regulate insulin signaling, thereby improving hunger tolerance and regulating metabolism and growth, enabling the body to respond to environmental challenges (147). The study found that NLaz is not important for immune resistance to gram-positive *E. faecalis* infection, but another lipocalin, Karl, released from blood cells, helps the host resist *E. faecalis* infection (147).

### 5.3 Eiger

Eiger is the homolog of tumor necrosis factor (TNF) in *D. melanogaster*, and members of the TNF family are important pleiotropic cytokines that play important roles in regulating infection, inflammation, autoimmune disease, and tissue homeostasis (148, 149). Evidence show that Eiger affect immune responses and metabolism and is a link between immune activation and systemic metabolism (128, 150, 151). Under low-protein diet conditions, Eiger is released from the fat body into the hemolymph and then into the brain, where it inhibits Dilp2 and Dilp3 expression by activating JNK signaling in IPCs by binding to the tumor necrosis factor receptor (128). Under immune stimulation, Eiger can be expressed in both blood cells and fat bodies, and influence the immune response under infection. The release of Eiger from the fat body under the condition of low protein diet can inhibit the expression of Dilp2-3 in IPCs (128), and the release of Eiger possible has the same effect under the condition of infection, which can change the body from anabolism to anti-infection by inhibiting IIS. Eiger expression in the fat body has a positive effect on host survival against extracellular pathogen infection, but loss of Eiger improves host survival when infected with intracellular pathogens, and this difference may stem from Eiger being required for phagocytosis (152, 153). Tang et al. showed that infected housefly *Musca domestica* by *Escherichia coli* or *Staphylococcus aureus* increased Eiger like gene (Mdeiger) expression resulting in decreased mortality, and knockdown of Mdeiger expression by RNAi downregulate expression of JNK and Dorsal, but upregulate the expression of Relish (154). These studies demonstrate that Eiger can enhance Toll-mediated immune responses, but in contrast to the IMD-Relish-mediated immune response, Eiger inhibits Relish activity by activating JNK, a result that is consistent with the Eiger mutant producing more IMD-regulated AMPs than wild-type (153, 154).

### 5.4 JAK

Cytokine Unpaire-dependent (Upd) activation of JAK-STAT signaling in *Drosophila* is similar to JAK-STAT signaling activated by type 1 cytokines such as IL-6 in mammalian systems (155, 156). In *D. melanogaster* adults, a high-fat diet stimulated blood cells to secrete Upd3, which in turn activated JAK-STAT signaling in muscle and gut and caused decreased insulin sensitivity (156). Deletion or specific silencing of the Upd3 gene in blood cells reduces JAK-STAT activation and increases insulin sensitivity and lifespan of *D. melanogaster* under high-fat diet conditions, while fat storage is not affected (156). The release of Upd from blood cells on a high-fat diet can cause decreased insulin sensitivity in peripheral tissues, similar to the role of its mammalian homolog, the proinflammatory cytokine IL-6, in insulin resistance (157, 158). Previous research showed that activation of JAK-STAT in muscle by cytokines Upd2 and Upd3 released from blood cells helps *D. melanogaster* to resist parasitic wasp infection (159). At the same time, this parasitic wasp infection can cause decreased insulin signaling in the muscle and fat body (160). However, the relationship between JAK-STAT and IIS is not well understood. The study on high-fat diet seems to provide insights into the relationship between Upd-JAK-IIS under infection condition (156), that is, under infection of parasitic wasps, Upd3 released by blood cells may reduce insulin sensitivity of the muscle and fat body by activating JAK/STAT, thus reducing the anabolism of the muscle and fat body and saving energy for blood cell’s cellular immunity to against parasitic wasp infestation. Agaisse et al. found that bacterial infection can also trigger blood cell-specific expression of Upd3, which in turn activates TotA-mediated fat body immune responses via JAK-STAT-Rrelish (161). However, specific downregulation of the JAK-STAT pathway transcription factor Stat92E in the fat body did not affect the body’s triglyceride storage, suggesting that JAK-STAT does not autonomously regulate energy metabolism in the fat body (162). The roles of Upd cytokines and JAK-STAT on insulin signaling and their relationship with metabolism in *Drosophila* immune response remain to be further elucidated.

### 5.5 Others

As the main component of insect blood sugar, trehalose is synthesized by trehalose-6-phosphate synthase (TPS) and trehalose phosphatase in insect fat body. Studies in houseflies *Musca domestica* show that trehalose-6-phosphate synthase (TPS) is involved in immune defense against pathogens by regulating the synthesis of trehalose (163). Studies have shown that systemic infection of pathogenic bacteria *E. coli* or *Staphylococcus aureus* can cause the increase of TPS transcription and trehalose content in the host, and the reduction of TPS content by RNA interference can lead to the increase of mortality of the host under infection condition, and this phenotype can be partially compensated by feeding trehalose (163).

Female parasitic wasps introduce various virulence factors into host insects during oviposition and realize parasitism by influencing host immunity and physiology. Recent studies have shown that female parasitic wasps *Pachycrepoideus vindemiae* use a novel venom protein, glucose-6-phosphate dehydrogenase (PvG6PDH), which affects carbohydrate metabolism by inhibiting the activity of glucose-6-phosphate (G6P) in host *Drosophila* to help achieve parasitism (164). However, the effect of PvG6PDH on the immune response of host insects during parasitism needs further study.

**Figure 2:**
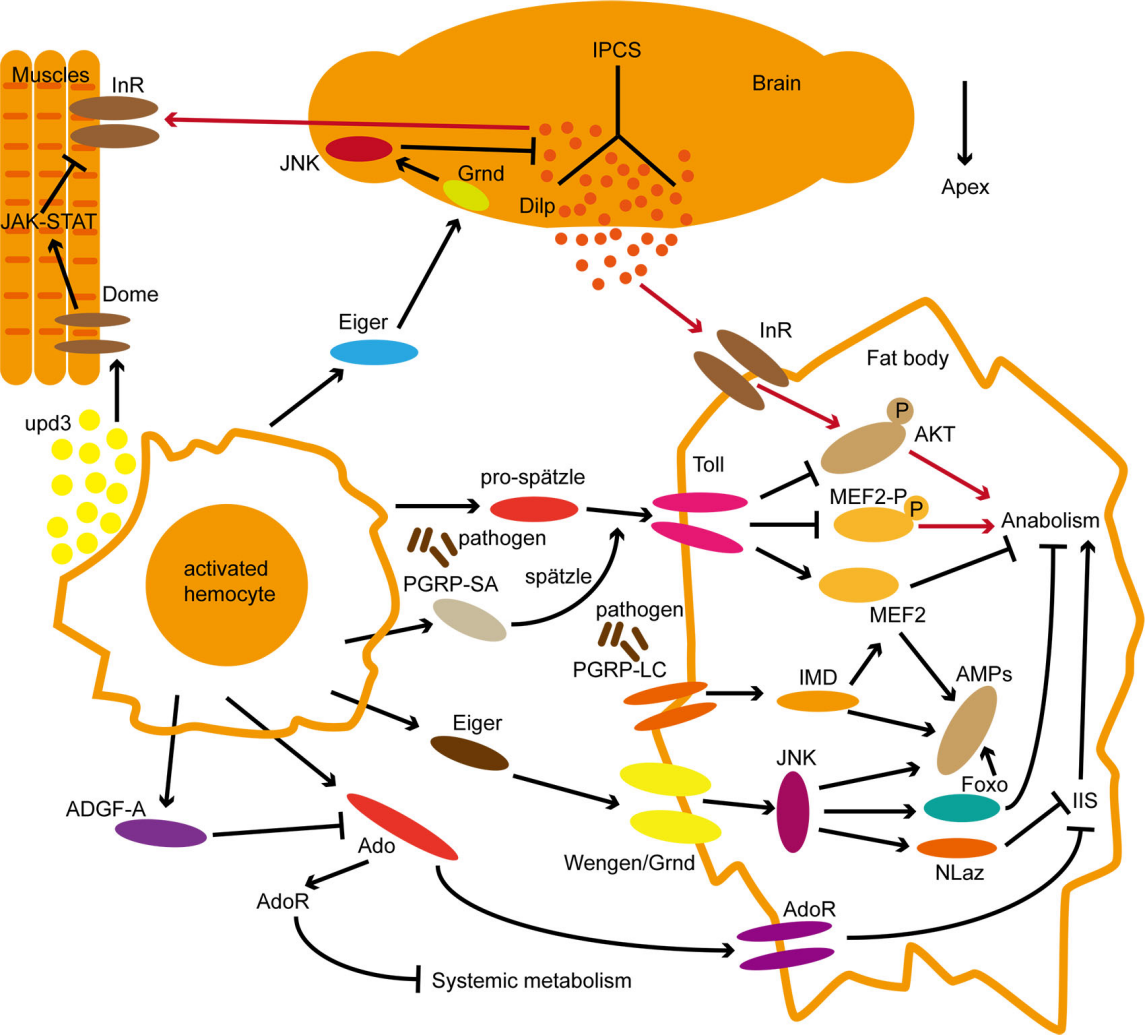
Systematic molecular regulation of immune and metabolic pathways in Drosophila. The red line represents metabolic regulation under normal conditions; The black lines represent signal regulation in the state of immune activation. Within pathways activity regulation, the arrow represents facilitation and the Bar represents inhibition roles. Refer to the text for the interaction of each pathway.

The fat body is an important site for energy reserves in insects, and infection with *Mycobacterium marinum* leads to progressive depletion of fat and glycogen in *Drosophila*, in part due to systemic AKT inactivation and FoxO dysregulation (14). Metabolic depletion phenotype similar to that described above after infection with *Listeria monocytogenes* (11). These metabolic phenotypes imply that the occurrence of systemic immunity will have an important impact on the body’s energy metabolism. These studies of core immune pathways demonstrate a direct link between metabolism and immune responses in the fat body energy redistribution at the molecular level (**Figure 2**).

## 6 Hemolymph

### 6.1 Hemolymph immune cell types and functions

In contrast to mammals which contain the closed circulatory system, the body cavity of insects is an open space filled with hemolymph (165). Insect hemolymph contains hemocytes and plasma, hemocytes are mainly macrophage-like plasmatocytes, which are involved in the cellular immune response (166). Insect hemocytes can be classified according to functional characteristics. In *D. melanogaster* hemocytes can be divided into three types: (1) plasmatocytes, the most abundant cell type in hemolymph, have phagocytosis and further differentiate into two other subtypes; (2) crystal cells that produce phenoloxidase (PO) and are involved in melanization; (3) Lamellocytes, flat adherent cells responsible for encapsulation of large foreign particles (16, 167–170). In some insects, granulocytes have the ability to adhere to foreign surfaces, similar to the Lamellocytes of *D. melanogaster*; in addition, spherulocytes have been found to transport components of the stratum corneum (171).

In *D. melanogaster* larvae, about 10% of the body’s glucose is needed to maintain quiescent hemocytes, and when cellular immunity is triggered, such as phagocytosis, the hemocytes expends more energy (8, 172). Immunometabolism mechanisms in vertebrate blood cells is not the focus of this review, and have been extensively discussed elsewhere (173–175). It is well understood that activated immune cells often need to switch from fatty acid oxidation to glycolysis, a phenomenon known as the Warburg effect, providing energy for rapid cellular proliferation and synthesis of antimicrobial effectors (173, 174). Once the physical barrier such as insect skin or intestinal membrane is compromised, and pathogens enter into insect body cavity, blood cells eliminate the invading pathogens through encapsulation, phagocytosis, nodulation, and melanization (176).

### 6.2 Immune cells and immunometabolism interactions

#### 6.2.1 Mammalian immune cells

To gain a clear understanding of cellular immunity in insects, it is necessary to understand the metabolic reorganization of mammalian immune cells. In mammals, when immune cells are activated, it results in a metabolic switch within immune cells that is dependent on the supply of large amounts of glucose and glutamine (177). When immune cells are inactivated, they use glycolysis to produce pyruvate which enters the mitochondria and converted to aceytyl-CoA. Acetyl-CoA is also produced by β-oxidation of fatty acids used in the tricarboxylic acid (TCA) cycle linked to oxidative phosphorylation (OXPHOS) to generate ATP in the most efficient manner. Thus, glycolysis and β-oxidation synergies with oxidative phosphorylation (OXPHOS) to produce ATP for energy supply to quiescent immune cells (178). While this is a very metabolically efficient form of ATP production, the rate at which is too slow to meet cellular energy demands in an immune-activated state. Upon immune stimulation, immune cells switch from a state of low nutrient uptake to an optimized metabolic state, rapidly producing ATP and synthesizing a large number of new molecules, ATP production is mainly through glycolysis, albeit not very efficiently (only 2 ATP per glucose molecule), but produces ATP much faster than the glycolysis-TCA-OXPHOS (43). Glucose carbons which produced through glycolysis are used to a greater extent by activated immune cells to produce new immune-effector macromolecules, and are less lost as carbon dioxide (CO_2_) through the oxidative phosphorylation pathway (OXPHOS). The pentose phosphate pathway (PPP) is a branch of glycolysis that generates ribose for nucleotide synthesis and NADPH for ROS (179–181). But mitochondrial metabolism is significantly altered in activated immune cells, pyruvate is diverted from the TCA cycle, which is disrupted in the citrate step, and glutamine is metabolized by the interstitium in mitochondria, replenishing TCA intermediates, thus become another important metabolite of activated immune cells (181, 182). In short, massively increased glycolysis (supplying PPP, lipid synthesis, and leading to lactate production) following immune cell activation is associated with rewired mitochondrial metabolism (breaking the TCA cycle and inhibiting OXPHOS), which makes activated immune cells dependent on high-dose glucose and glutamine supply (177).

#### 6.2.2 Metabolic remodeling in insect blood cells

Metabolic reprogramming of activated immune cells has been studied primarily in mammalian systems, and although the bactericidal function of insect macrophages (hemocytes) is highly conserved with mammals, the metabolic remodeling of insect macrophages remains poorly understood. Nevertheless, the Warburg effect has also been found in insect cancer cells as well as in activated immune cells (183–186). Myc is an important regulator of immune cell metabolic remodeling, and in mammals, the metabolic reorganization of proliferating T lymphocytes is associated with Myc activation (187, 188). In insects, Myc is strongly expressed in blood cells of the highly proliferating Hop lethal mutant line (hop tuml) (189). Hypoxia-inducible factor 1α (HIF-1α) was originally found to regulate cellular metabolism by participating in mitochondrial oxidative phosphorylation under hypoxia (190). In mammals, activation of Toll-like receptors and NF-κB signaling leads to increased expression and enhanced stability of HIF-1α (191, 192). Krejčová et al. used adult *D. melanogaster* to study the metabolic changes of macrophages during the acute and remission phases of *Streptococcus*-induced sepsis and found that macrophage activation, bactericidal and fighting infection depends on the help of HIF-1α and lactate dehydrogenase homologues (193). This demonstrate the existence of a cellular metabolic mechanism in insects that is conserved with mammals, namely that macrophages induce systemic metabolic changes through aerobic glycolysis (193). Metabolic pathway studies during *D. melanogaster* plasmatocytes activation revealed that peroxisomes, an organelle related to lipid metabolism and oxidation reactions in plasmatocytes, are required for phagosome formation and antimicrobial peptide production, and this role conserved with mouse macrophages (194). Wasps such as *Leptopilina boulardi* parasitize *D. melanogaster* larvae by laying eggs that are too large to be phagocytosed (195). The *D. melanogaster* kill the invading parasite’s eggs by encapsulation them with lamellocytes, a type of differentiated hemocytes (196). During parasitoid infestation of *Drosophila* larvae, lamellocytes proliferation and differentiation are associated with increased blood cell-specific glycolytic gene expression, accompanied by increased glucose consumption and lactate production (8). In addition, similar findings have been made in other insects. For example, activation of phagocytes in *Blaberus giganteus* produces metabolic changes similar to the Warburg effect (172). The transcriptome analysis of immune-activated hemocytes in mosquitoes and *Spodoptera exigua* also revealed associations with glycolytic and LDH genes (150, 197–200). At present, studies based on a variety of insects have found that activated immune cells can significantly increase glucose consumption, glycolysis and lactic acid production, but the relationship between insect immunity and metabolism is still not as clear as that of mammals, and further investigation is needed.

During the immune response, metabolic regulation ensures that sufficient energy is available for an effective immune response, but since energy is finite, so immune responses must be properly regulated to accommodate the energy demands of other physiological characteristics. Like many immune responses, activation of lamellocyte requires reallocation of resources to fuel the differentiation and activation of blood cells (7). To this end, blood cells secrete extracellular adenosine (e-Ado), which enables other tissues to release stored glucose to provide energy for the activation of lamellocyte (8). However, the release of e-Ado must be regulated because the increased energy (glucose) of the blood body cavity is also available to pathogens. Later in infection, immune cells express the adenosine deaminase (ADGF-A) to regulate e-Ado levels and prevent pathogens from exploiting host resources (9). Interestingly, high levels of e-Ado were detected in human sepsis, suggesting that e-Ado has a similar effect in mammals (201, 202). Lin et al. found that adenosine receptor signaling (AdoR) is involved in the regulation of metabolic remodeling after budding virus (AcMNPV) infection in silkworm *Bombyx mori*, which can promote virus clearance and antiviral protein gloverin production (203). The study on another lepidopteran insect model *Spodoptera frugiperda* Sf-21 cells proved that in the metabolic regulation of Sf-21 cells after infection with baculovirus, adenosine signal transduction activates the host’s energy synthesis, supporting the innate immune response against infection, showing that glycolysis regulated by adenosine signaling pathway is a conserved mechanism (203). Another study on the regulation of adenosine metabolism showed that the symbiotic virus (SmBV) of the parasitic wasp *Snellenius manilae* was able to downregulate the extracellular adenosine (e-Ado) of the host *Spodoptera litura*, thereby inhibiting host metabolic switching and attenuating its immune response (204). This study provides us with a new perspective that the parasitoid symbiotic virus regulates the host’s adenosine pathway, allowing the eggs and larvae of *S. manilae* to evade the immune response of the host *Spodoptera litura* (**Figure 2**).

PGRP-SA and Spätzle, produced by hemocytes, are critical for the activation of Toll in fat, and their expression increased in response to pathogen infection (129, 205, 206). Because Toll activation leads not only to the expression of AMPs but also to the inhibition of insulin signaling, it implies that blood cells play a role in the activation of humoral immunity associated with metabolic switches through the expression of PGRP-SA and Spätzle. Cytokines released by blood cells have also been implicated in the control of metabolic homeostasis in *D. melanogaster* raised on a high-fat diet increased the expression of the Upd3 cytokine in blood cells, leading to systemic activation of JAK/STAT and decreased sensitivity to insulin (207). Under infection conditions, blood cells can not only participate in immune regulation but also regulate body metabolism by releasing cellular factors such as Eiger and Upd (150, 159).

#### 6.2.3 Immunometabolic regulation of insect hemolymph symbionts

The microbiota in insects is controlled by the host, i.e. the abundance, composition and location of microorganisms must all be within a certain range (208). For microorganisms, hemolymph is rich in nutrients, has a balanced ionic composition, and has a PH value close to neutral, which is very beneficial for their survival (208). However, the hemolymph is protected by the immune system, including blood cells and a variety of soluble effectors (antibacterial peptides, reactive oxygen species, phenoloxidases, etc.) that can kill, phagocytose, and encapsulate invading microorganisms to varying degrees (33). For a long time, research believed that in healthy insects, the hemolymph had little or no microorganisms (33). But recent studies have shown that various non-pathogenic microorganisms can stably or transiently inhabit within the hemolymph of a wide variety of insects (209). The most widely reported hemolymph microorganisms in insects are *spiroplasmas* of firmicutes, and these bacteria are mostly distributed in Hymenoptera, Hemiptera, Lepidoptera, Coleoptera and Diptera (209–211). *Spiroplasmas* are mainly found in the hemolymph or intestinal lumen, but can also be present in the fat body and ovary to cause intracellular infections (212). For example, the bacteria of *spiroplasma* in the hemolymph of *D. melanogaster* combine with yolk granules released from adipocytes and are transformed into developing oocytes through hemolymph to achieve vertical transmission (213). Moreover, a large number of bacterial members of the enterobacteriaceae (gamma-proteobacteria) are present in the hemolymph of insects, especially the three symbiotic bacteria found in aphids: *Serratia Symbiotica* and *Hamiltonella defensa* and *Regiella insecticola*, also known as secondary symbiotic bacteria (209). In some insects, the hemolymph microbiome also includes obligate intracellular symbiotic bacterial groups, such as *Wolbachia* and *Rickettsia* of the order Rickettsiales (alpha-proteobacteria), which are very common in insects (214, 215).

MAMPs of bacterial cell wall are target molecules recognized by insect immune receptors, and the interaction between hemolymph symbionts and the immune system depends on the extent of bacterial genome alterations. Genome sequencing of insect symbionts revealed that they suffer deletions in their gene sequences including virulence genes and bacterial cell wall element genes (216–219). Gene expression profiling of *D. melanogaster* with and without *spiroplasma* found no significant differences in the expression of various immune-related genes, including those expressing antimicrobial peptides (212, 220, 221). The reason behind this could be that *Spiroplasma* lacks the peptidoglycan cell wall of most bacteria and thus is not recognized by the host’s pattern recognition receptors to activate the insect’s immune response (33). Analysis of the hemolymph of *D. melanogaster* colonized with and without *Spiroplasma* showed increased amino acid concentrations and decreased lipid titers (triacylglycerol TAG and Diacylglycerol DAG), however, sugar and sterol levels and storage of the main carbohydrate glycogen were not significantly altered (213). This study further demonstrated that these host metabolic differences are mediated by *Spiroplasma* consumption of DAG, and reduced the production of insect lipoproteins (lipoproteins that transport DAG in hemolymph) by RNAi can significantly reduce the amount of DAG and *Spiroplasma* populations in insect hemolymph; Furthermore, in mutant flies that block fat mobilization from the fat body (specifically double mutations in the Brummer lipase and adipokinetic hormone receptor genes), *Spiroplasma* titers are not affected (213). These results suggest that DAG utilized by *Spiroplasma* are truncated dietary-derived lipids rather than stored lipids mobilized from the fat body and other organs. *Spiroplasma* has been shown to protect *Drosophila melanogaster* and *Drosophila hydei* from parasitic wasps (222, 223). Additionally, *Spiroplasmas* also protects *Drosophila neotestacea* from parasitic wasps and nematodes (*Howardula aeronymphium*), protecting the pea aphid (*Acyrthosiphon pisum Harris*) from pathogenic fungi (222–225). Among them, the protective effect of *Spiroplasmas* on host from parasitism was attributed to the influence of host metabolism, especially the competitive utilization of DAG by *spiroplasmas* in *D. melanogaster* (226).

To understand the metabolic effects of symbionts in host hemolymph of pea aphid, a comparative metabolomic analysis was conducted between aphid with secondary symbionts (*Serratia symbiotica, Hamiltonella, Regiella)* and without them (227). The results showed that there were significant differences in metabolites between the aphid with and without symbiosis, but the metabolomics characteristics of the aphid with different species of secondary symbiosis were highly similar, and the levels of amino acids in aphid samples containing secondary symbiotic bacteria increased compared to controls, while the levels of sugars and sugar alcohols decreased (227). These results suggest that the hemolymph symbiotic bacteria can affect host metabolism of aphid. Research on the effects of secondary symbiotic bacteria on the immune system of aphid mainly focused on cellular immune response. Laughton et al. used *Serratia symbiotica*, *Hamiltonella defensa* and *Regiella insecticola* to colonize aphid respectively, demonstrating increased hemocytes density in hemolymph samples from aphid colonized with *Hamiltonella defensa* and enhanced hemocytes response of encapsulation to Sephadex beads from aphid hemolymph colonized with *Regiella insecticola*, suggesting that the innate immune system of host insects can recognize these hemolymph symbiosis bacteria (228, 229). These findings suggest that the symbiotic bacteria in aphid hemolymph can affect the metabolism and immunity of the host, but whether there is a direct link between immune-metabolism remains to be further investigated.

*Wolbachia* were first identified in the mosquito’s germline over a century ago, and as an endosymbiont, they are surprisingly diverse and can co-exist with up to 66% of insect species (230). It can be stably inherited in the host population and influence the evolution, immunity, physiology and development of the host (231). Three major surface proteins have been identified in *Wolbachia*: wsp and its two analogs, wspA and wspB, which are associated with important pathogenic bacteria such as *Ehrlichia* and *Neisseria* membrane proteins with antigenic function are homologous (232–234). Studies in vertebrates have shown that after infection with *Wolbachia*-containing Filarial nematodes, the host can elicit an immune response by recognizing the *Wolbachia* membrane protein wsp (235), suggesting that *Wolbachia* may play a role in redirecting immune responses in vertebrates. In *D. melanogaster*, *Wolbachia* infection is widespread. Research has compared the differences in IIS-related phenotypes in the presence and absence of *Wolbachia* in IIS mutant flies. They show the absence of *Wolbachia* in IIS mutant flies further reduces IIS activity and indicating the role of *Wolbachia* in normal could increase host IIS activity and promote grow of host flies (236). In conclusion, the presence of the endosymbiotic *Wolbachia* can improve the IIS activity of the host, contrary to the influence of the pathogenic pathogen on the host IIS activity (236). Meanwhile, studies on the *Wolbachia* surface protein Wsp in vertebrates also showed that the *Wolbachia* can induce the host immune response (235).

## 7 Regulation of immunometabolism in other tissues

Muscle is a major energy consuming organ, Zhao et al. found that feeding Pe (*Pseudomonas entomophila*) bacteria can induce differential expression of NF-κB signal in epidermal muscle tissue between individual *D. melanogaster* populations (237). *D. melanogaster* with restricted expression of NF-κB/IMD signal not only exhibited longer life span, but also showed increased defecation phenotype. In these individual’s muscle tissue, glutamate dehydrogenase (Gdh) expression is significantly upregulated in NF-κB signaling was mildly activated, leading to an increase in circulating glutamate content; glutamate can participate in vitamin metabolism mediated by sodium-dependent multivitamin transporter (svmt) after transported by glutamate transporter (dmGlut) into adipose tissue, ultimately leading to enhanced lipid mobilization and intestinal defecation response (237). These findings suggest that modest activation of NF-κB signaling in muscle tissue contributes to improved host survival under oral infection by mobilizing lipolysis in the fat body. Zhao et al. showed that oral bacterial infection can remotely regulate immune and metabolic reactions in the muscle and fat body to help the host eliminate bacteria (237), suggesting that such communication between organs is crucial to host homeostasis regulation under infection. Yang et al. also found multi-organ communication under oral bacterial infection, the IMD response of *D. melanogaster* is activated sequentially from the gut to the fat body under the mediating of the polyol pathway of sugar alcohols (238). The gut IMD activation leads to an increase in sorbitol concentration in the hemolymph, which in turn activates metalloproteinase 2 (Mmp2), and the activated Mmp2 cleaves PGRP-LC to activate the IMD response in the fat body (238).

## 8 Conclusion

Insects go through different developmental stages such as eggs, larvae, pupa, and adults throughout their lives. The interaction between microorganisms and insects exists in different developmental stages. Therefore, the coordinated regulation of immunity and metabolism is essential for the normal growth, development, and survival of insects. Toll, Imd, Eiger/TNF-α, JNK and JAK-STAT are the most important signals in the immune response, and they are activated by various immune stimuli. In addition to being critical for inducing immune responses, they also affect metabolism at various levels. Although immune-metabolism research in other invertebrates have not been extensively and comprehensively carried out, some existing examples are listed here (**Table 1**). At the same time, we summarized the molecular regulatory networks of immunity and metabolism in existing studies (**Table 2**).

**Table 1:** Immune-metabolic interactions in insects.

Host	Microorganism/ Parasites	Location in hosts	Role	Immune phenotype of hosts	Metabolic phenotype of hosts	Reference
*Sitophilus zeamais* (Coleoptera: Curculionidae)	*Sitophilus* primary endosymbiont (SPE)	Bacteriocytes Oocytes	Endosymbionts	AMP-coleoptericin A (ColA) (+)	Nutrient acquisition (-); Energy metabolism (-)	(239, 240)
*Aphis gossypii* Glover (Hemiptera: Aphididae)	*Lysiphlebia japonica* Ashmead (Hymenoptera: Braconidae)	Hemocoel	Endoparasitic	Serine protease; SerpinB (+); Two SOD (+); Two lectins (+); Two galectins (+)	Biosynthes of proteins (+); Glutamine metabolism (+); Energy and carbohydrate production (+); Lipid metabolism (+)	(241)
*Helicoverpa armigera* (Lepidoptera: Noctuidae)	*Microplitis mediator* (Hymenoptera: Braconidae)	Hemocoel	Endoparasitic	Immune response (+)	Carbohydrate, fatty acid, amino acid metabolism (6 h post-parasitism) (+)	(242)
*Pieris rapae* (Lepidoptera: Pieridae)	*Pteromalus puparum* (Hymenoptera: Pteromalidae)	Hemocoel	Endoparasitic	Immune response (+)	Alanine, aspartate, glutamate, starch, sucrose, arginine and proline metabolism pathway in hemolymph of host were changed after PpAmy3 injection.	(243)
*Bombyx mori* (Lepidoptera: Bombycidae)	*Bombyx mori* nucleopolyhedrovirus (BmNPV)	Systemic infection	Pathogenic microorganisms	Be killed	Expression of *BmFoxO* (-)	(244)
*Plagiodera versicolora* (Coleoptera: Chrysomelidae)	Gut microbiota	Gut	Endosymbionts	Immunity-related genes (peptidoglycan-recognition protein, defensin, and prophenoloxidase) in larvaes gut (+)	The glycometabolism metabolism in larvae (-)	(245)
*Hermetia illucens* (Diptera: Stratiomyidae)	*Escherichia coli* and *Staphylococcus aureus*	Systemic infection	Pathogenic Microorganisms	Systemic immune response (+)	Alanine, aspartate, glutamate, arginine, proline, purine and pyruvate metabolism were impacted.	(246)
*Diaphorina citri* Kuwayama (Hemiptera: Psyllidae)	*Candidatus* Liberibacter asiaticus (CLas)	Alimentary canal/ Salivary glands/ hemolymph/ Filter chamber/ Midgut Fat body Muscle Ovary	Vector of CLas	Synthesize and release immune-related proteases (-)	The vitamin B6, glycerophospholipid and purine metabolism pathway (+); The pantothenate, CoA biosynthesis, cysteine and methionine metabolism pathways (-)	(247, 248)
*Glossina fuscipes fuscipes* (*Gff*) (Diptera: Muscidae)	*Spiroplasma*	Reproductive Digestive tissue Hemolymph	Endosymbionts	Resistance to infection with trypanosomes (+); Toll pathway activity in the male gonads (+)	Circulating level of TAG in females hemolymph (-)	(249)
*Aedes fluviatilis* (Diptera: Culicidae)	*Wolbachia pipientis*	Oocytes	Endosymbionts	IMD and Toll pathways activity in the ovaries post blood meal (+)	Proteolysis and cytosolic PEPCK transcript levels in the ovaries between 24 h and 48 h post blood meal (+)	(250)
*Anopheles stephensi* (Diptera: Culicidae)	*Plasmodium falciparum*	Midgut	Vector of *P. falciparum*	p38-MAPK pathway activity (+); The production of mitochondrial reactive oxygen species (-)	Mitochondrial biogenesis, oxidative phosphorylation (OXPHOS), antioxidant biosynthesis, and protein translation (+)	(71)
*Bombyx mori* (Lepidoptera, Bombycidae)	*Autographa californica* nucleopolyhedrovirus (AcMNPV)	Systemic infection	Pathogenic Microorganisms	The production of the antivirus protein and gloverin (+)	Adenosine signaling activity (+); The level of ATP production(+); The level of hemolymph glucose at 48 h post infection (-).	(203)
*Spodoptera litura* (Lepidoptera, Noctuidae)	*Snellenius manilae* bracoviruses, SmBVs	*Snellenius manilae* injected egg and SmBVs into hemocoel of *Spodoptera litura*	SmBVs is symbiont virus of *S.manilae; S.manilae* is an endoparasitic wasp of *Spodoptera litura*	SmBVs inhibited the expression of immune gene of *S. litura.*	SmBVs inhibited the content of the extracellular adenosine of *S. litura*; Glycolysis and carbohydrate mobilization in the hemocyte of infected larvae were inhibited; Carbohydrate mobilization, glycogenolysis, and ATP synthesis (-).	(204)

“+” represents up-regulated; “-” represents down-regulated.

**Table 2:** The immunity and metabolism networks of host.

Species	Immune signaling	Metabolic signaling	Mechanism	Reference
Pathogenic bacteria	Toll/IMD	IIS	Toll ⊣ AKT ⇥ Foxo → AMPs /catabolism	(130)
		Hippo	Toll → Pelle → Dif → AMPs	(141)
			Toll → Pelle →WTS ⊣ Yorkie	
		MEF2	Health condition: S6K → MEF2-P → anabolism	(131, 132)
			Stimulation: IMD/Toll ⊣ S6K ⇒ MEF2-TBP ⊣ anabolism	
			IMD/Toll ⊣ S6K ⇒ MEF2-TBP → AMPs	
		TK	PGRP-LC → IMD ⊣ TK → Lipogenesis	(75)
	ROS/Duox	TRAF3	TRAF3 ⊣ AKT ⊣ TOR ⊣ S6K	(69)
			TRAF3 → AMPK ⊣ TOR → ATG1 → Catabolic	
			P38-MAPK → Protein synthesis	(71)
			P38-MAPK ⊣ ROS	
Nonpathogenic bacteria	IMD	IIS	Acetate → IIS	(99)
			Acetate → PGRP-LC → Relish → TK ⊣ Lipid Accumulation	(105, 107)
		TOR	*Lactobacillus plantarum* → IMD → Intestinal peptidase → TOR	(90, 91)
	ROS/Duox	MKP3	MKP3 ⊣ P38-MAPK → Low-level Duox	(121)

“→” represents activation; “⊣” represents repression; “⇥” represents derepression; “⇒” represents induction.

Although current research has clarified the relationship between immunity and metabolism, the specific physiological functions of the interaction between the two, the differences in the immune-metabolism interaction between different sexes of insects, the effect of the brain on vertebrates and invertebrates between different organs and signal coordination remains to be further explored (69). The brain has extensive regulation of the body’s eating behavior and metabolism (251, 252), and studies have also confirmed the coordination of the brain’s immune response (253). Therefore, the regulation of the brain’s immunity and metabolism cannot be ignored. The advancement of technology makes personalized medicine possible; the above research issues should be paid more attention in future research. Insect, as an ancient and simple research model, especially *D. melanogaster* is a convenient means of genetic manipulation, so that we can use insect model research, not only to provide guidance for the study of immunometabolic diseases in vertebrates including humans, but also to provide strategies from the perspective of immunometabolism for the control of agricultural insect pests. The more hypotheses and proposals for future are shown as the **Table 3**.

**Table 3:** The hypotheses and proposals for future in our review.

Hypotheses and proposals for future
• It is necessary to further investigate the distribution and diversity of microbial species in insect hemolymph.
• Investigate the correlation between the abundance and proliferation rate of hemolymph microbes and the nutritional status and immune response intensity of insects.
• Investigate the effects of hemolymph microorganisms on core metabolic pathways such as insulin signaling pathways of host insects.
• Identify the molecular mechanisms of interactions between specific effectors and host receptors in bacteria and determine how these interactions translate into effects on host insect immunometabolism.
• In addition to the core metabolic pathways, the convenient genetic manipulation techniques of *Drosophila* can be used to further explore the influence of specific microbial effectors in specific downstream metabolic pathways.
• The mechanism of microbial regulation of host immunometabolism in other insects also needs to be further studied, providing reference for a more comprehensive understanding of the effects of microorganisms on host physiology and biochemistry.
• Sex as an important factor should be given more attention in the future about immunometabolic studies.
• With the advancement of technology, it is more beneficial to explore the systematic coordination mechanism of immune metabolism regulation within and between tissues by using cell sorting and metabolomics etc. technology in the future.
• Future immunometabolic studies should coordinate the regulation mechanism between nervous system with other tissues in both vertebrates and invertebrates.

## Author contributions

SL, JW and XT were responsible for the study concept, design, and writing of the first draft. XT designed and drew the Figure and Table. ST provided literatures review and responsible for modify the content of the article. WH reviewed, proofread, and revised the draft. All authors contributed to the article and approved the submitted version.
